# Invasive Breast Carcinoma of No Special Type with Medullary Pattern: Morphological and Immunohistochemical Features

**DOI:** 10.5146/tjpath.2021.01559

**Published:** 2022-09-15

**Authors:** Mykola Lyndin, Nataliia Hyriavenko, Vladyslav Sikora, Yuliia Lyndina, Yuliia Soroka, Anatolii Romaniuk

**Affiliations:** Department of Pathology, Medical Institute, Sumy State University, Sumy, Ukraine; Department of Fifth-Year Student, Medical Institute, Sumy State University, Sumy, Ukraine

**Keywords:** Breast carcinoma, Triple negative breast cancer, Medullary carcinoma, Tumor microenvironment, Tumor infiltrating lymphocyte

## Abstract

***Objective:*** Our study investigated the morphological and immunohistochemical characteristics of invasive breast carcinoma of no special type (IBC-NST) with medullary pattern to explore the inconsistencies between the structural and clinical traits of this category of tumor.

***Material and Method:*** The breast carcinoma samples (n = 26) with medullary pattern (defined according to established criteria) were subjected to immunohistochemical assays of the following receptors: ER, PR, HER2/neu, Ki-67, p53, Bcl-2, VEGF, MMP1, E-cadherin, EGFR, Hsp70, Hsp90, CD20, CD3, CD4, CD8, CD68, CD163, CD56, CD138, MPO, S100, IgG, IgM, and PD-L1.

***Results:*** IBC-NST with medullary pattern was found to have negative expression of ER, PR, and HER2/neu; strong positive expression of Kі-67, mutant р53, Bcl-2, E-cadherin, EGFR, and PD-L1; moderate positive expression of Hsp70 and Hsp90; and low or negative expression of VEGF and MMP1. Furthermore, there was pronounced variability in the qualitative composition of tumor immune infiltrates with regards to T-lymphocytes, B-lymphocytes, macrophages, plasmocytes, and granulocytes.

***Conclusion:*** IBC-NST with medullary pattern has many unfavourable morphological and immunohistochemical prognostic characteristics, which are balanced against the pronounced protective properties of the tumor cells and the qualitative characteristics of the tumor microenvironment. These can lead to a favourable disease course despite the relatively adverse features of the carcinoma cells.

## INTRODUCTION

Breast carcinoma (ВС) is the most common malignancy worldwide, accounting for 11.7% of all cancer cases, with a prevalence of up to 24.5% among women. Despite detection being possible at increasingly earlier stages, the BC mortality rate remains relatively high (accounting for 6.9% of all oncological deaths among both genders) (1). The course of the disease, treatment sensitivity, and prognosis depend on the tumor histological type, grade, stage, vascular invasion, immunophenotype of neoplastic cells, and tumor microenvironment (2). Until recently, medullary carcinoma was considered the most favourable variant of BC (3). In the latest World Health Organisation (WHO) classification of breast tumors, published in 2018, this type of carcinoma was included in the category of invasive BC of no special type (IBC-NST). Now it has name IBC-NST with medullary pattern or tumor -infiltrating lymphocytes (TILs) rich IBC-NST (2,4).

IBC-NST is well-defined and has a syncytial growth pattern with no glandular structures of high histological grade as well as prominent TILs (2,5). Cases are mainly triple-negative without expression of the еstrogen receptor (ER), progesterone receptor (PR), and human epidermal growth factor receptor 2 (HER2/neu). Pronounced proliferative activity of tumor cells and expression of tumor SUPPRESOR protein р53 are commonly observed (5). However, despite the potentially unfavourable histological features, IBC-NST with medullary pattern has a relatively good prognosis (2,6,7); notably, this has been associated with the qualitative features of the tumor microenvironment (TILs) and pronounced tumor cell adhesion (involving E-cadherin expression) and absence of matrix metalloproteinase-1 (MMP1) (2,5,6).

The favourable course of this high-grade carcinoma has not been explained by the presence or absence of the abovementioned proteins. Further studies are required to explain the inconsistencies between the morphological and clinical features of IBC-NST with medullary pattern.

## MATERIALS and METHODS

### Patients and Samples

Cases of IBC-NST with medullary pattern (n = 26) were identified among patients who underwent mastectomy and sectoral resection of the breast from 2010 to 2020 at the Surgical Department of the Sumy Regional Oncology Centre in Ukraine. Samples were included in the study group if they met the following criteria: 1) well-defined tumor contours with a peripheral pseudocapsule; 2) syncytia formation in >75% of the tumor area; 3) absence of tubular and glandular tumor features; 4) pronounced lymphoplasmacytic stromal infiltration; 5) high histological grade; 6) absence of metastases; and 7) absence of ER, PR, and HER2/neu expression (2,5). Written informed consent for tissue investigation was obtained from all patients. The Bioethics Commission of the Medical Institute of Sumy State University approved the experimental protocol (no. 5/2 from 12.02.2017).

### Immunohistochemical Assay

Paraffin-block samples stored for histological diagnosis were used in the immunohistochemical assay following the method detailed in our previous publication (8). The antibody panels (purchased from Thermo Scientific, Master Diagnóstica, and Abcam) shown in Table 1 were employed to establish the parenchymal and stromal components of the immunophenotype of IBC-NST with medullary pattern.

**Table 1:** Antibody panel used for immunohistochemical study.

**Antibody**	**Host**	**Clone**	**Pattern**
ER*	Rabbit	SP1	Nucleus
PR*	Rabbit	YR85	Nucleus
HER2/neu*	Rabbit	SP3	Membrane
Ki-67*	Rabbit	SP6	Nucleus
p53*	Mouse	SP5	Nucleus
Bcl-2*	Mouse	100/D5	Cytoplasm
VEGF*	Rabbit	Polyclon	Cytoplasm
MMP1*	Rabbit	Polyclon	Cytoplasm
E-cadherin*	Rabbit	67A4	Membrane
EGFR*	Rabbit	EP38Y	Membrane
Hsp90*	Rabbit	Polyclon	Cytoplasm, nucleus
Hsp70*	Mouse	W27	Cytoplasm, nucleus
CD20*	Mouse	L26	Membrane, cytoplasm
CD3*	Rabbit	SP7	Membrane, cytoplasm
CD4**	Rabbit	EP204	Membrane, cytoplasm
CD8**	Rabbit	SP16	Membrane, cytoplasm
CD68*	Mouse	KP1	Cytoplasm
CD163**	Rabbit	EP324	Membrane, cytoplasm
CD56***	Mouse	123C3	Membrane, cytoplasm
CD138**	Rabbit	EP201	Membrane, cytoplasm
MPO*	Rabbit	Polyclon	Cytoplasm
S100*	Mouse	4C4.9	Cytoplasm
IgG*	Rabbit	Polyclon	Cytoplasm
IgM*	Rabbit	Polyclon	Cytoplasm
PD-L1**	Rabbit	CAL10	Membrane

Thermo Scientific*, Master Diagnóstica**, Abcam***

### Evaluation of Staining

Two pathologists independently analysed the immunohistochemical staining evaluations and reached a consensus decision when necessary. The reaction was considered positive if the cells had cytoplasmic and/or nuclear expression of heat-shock protein 70 (Hsp70) and 90 (Hsp90); cytoplasmic and/or membranous expression of CD20, CD3, CD4, CD8, CD163, CD56, and CD138; exclusively nuclear expression of ER, PR, Ki-67, and tumor SUPPRESOR protein p53; exclusively cytoplasmic expression of Bcl-2, VEGF, MMP1, CD68, MPO, S100, IgG, and IgM; and exclusively membranous expression of HER2/neu, EGFR, and E-cadherin. To determine the pattern of receptor expression, we used a simplified 3-tiered scoring system: “-” – negative expression (0% immunoreactive cells); “+” – low expression (5–25% immunoreactive cells); “++” – moderate expression (26–50% immunoreactive cells); “+++” – high expression (>50% immunoreactive cells). Only the p53 nuclear expression pattern was considered as positive or negative staining. Ki-67, Hsp70 and Hsp90 expression were scored in percentage. The percentage of receptor-positive cells among the total number of tumor cells was scored. The intensity of the stained cells was considered separately. PD-L1 expression was considered positive in the presence of more than 1% of carcinoma cells showing membranous staining of any intensity. This scoring sheet was discussed with and approved by two pathologists prior to the start of the study. No morphologic features of the cancer were taken into account in this scoring. Data processing was carried out using the GraphPad Prism 9 statistics software. Detection and evaluation of the links among indicators were carried out using the nonparametric Spearman’s rank correlation coefficient (r). A p-value of 0.05 (95% level of confidence) was considered statistically significant.

## RESULTS

**Figure 1:**
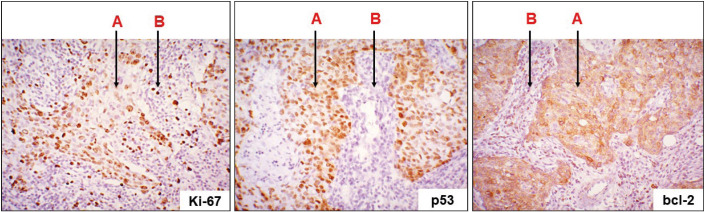
IBC-NST with medullary pattern: a pronounced expression of Ki-67, p53, and bcl-2 in tumoral cells (A); immune microenvironment cells (Ki-67- and bcl-2-positive expression) (B). Immunohistochemical study of Ki-67, p53, and bcl-2; x200.

The results of the immunohistochemical study showed that all cases of IBC-NST with medullary pattern were ER-, PR- and HER2-negative. At the same time, pronounced proliferative activity (nuclear Kі-67 expression in 78.4±4.6% of tumor cells), mutant р53 expression (nuclear expression in 92% of cases), and hyperproduction of anti-apoptotic Bcl-2 protein (cytoplasmic expression) were observed in all cases (Figure 1). Besides the neoplastic cells, some cells of the immune infiltrate of the tumor microenvironment were Kі-67- and Bcl-2-positive.

**Figure 2:**
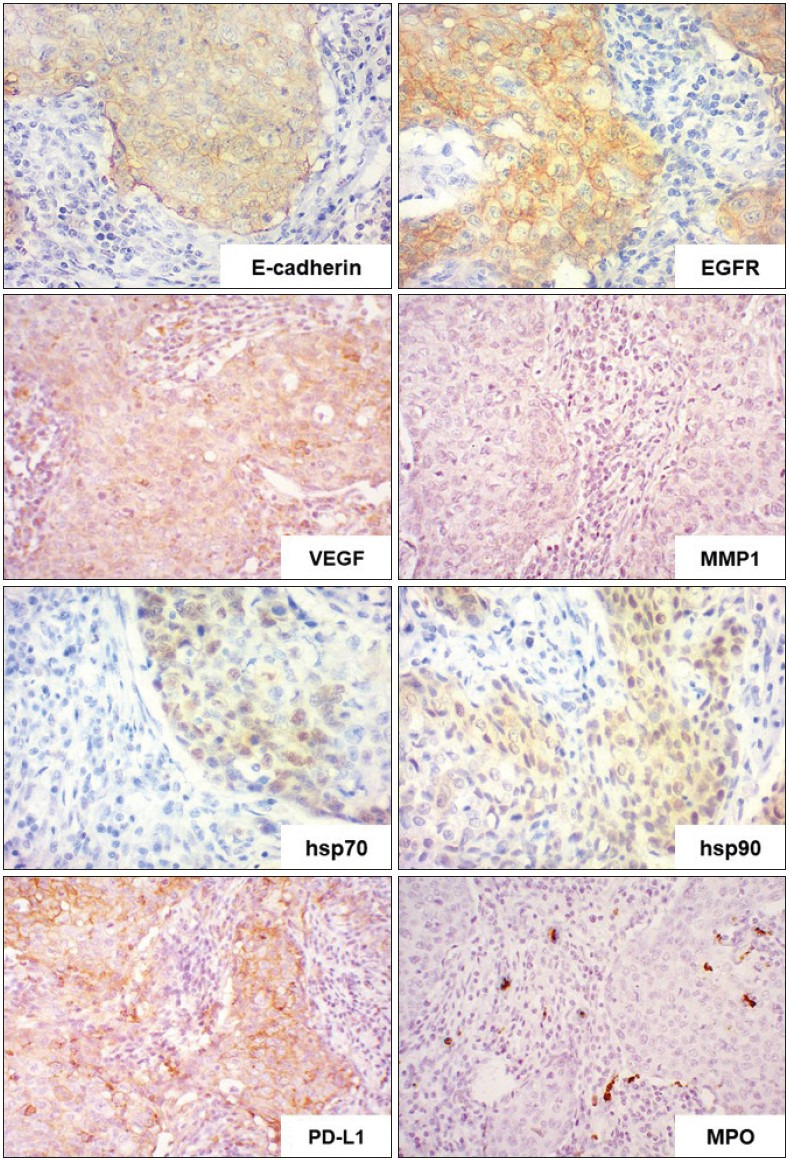
IBC-NST with medullary pattern: a pronounced expression of E-cadherin, EGFR, and PD-L1; moderate expression of hsp70 and hsp90; week expression of MMP1 and VEGF; negative expression of MPO in tumoral cells, positive in scattered cells of microenvironment. Immunohistochemical study of E-cadherin, EGFR, hsp70, hsp90, VEGF, MMP1, PD-L1, and MPO ; x400.

All cases of IBC-NST with medullary pattern had strong (immunoexpression in more than 50% of tumor cells) membranous expression of E-cadherin, EGFR, and PD-L1 in the cancer cells (Figure 2). Moderate positive nuclear and cytoplasmic expression of Hsp70 and Hsp90 was observed in 74±6.3% and 68±5.7% of the tumor cells, respectively. Simultaneously, the majority of the tumor cells were either negative or had low (weak expression in less than 25% of tumor cells) VEGF and MMP1 cytoplasmic expression (Table 2).

**Table 2:** Protein expressions in IBC-NST with medullary pattern.

	**ER**	**PR**	**HER2**	**Ki-67** **(% of cells)**	**P53**	**Bcl-2**	**E-cadherin**	**EGFR**	**PD-L1**	**Hsp70** **(% of cells)**	**Hsp90** **(% of cells)**	**VEGF**	**MMP1**
**1**	**–**	**–**	**–**	75	+	+++	+++	+++	+++	82	67	+	–
**2**	**–**	**–**	**–**	78	+	+++	+++	+++	+++	69	72	–	+
**3**	**–**	**–**	**–**	80	+	++	+++	+++	+++	63	77	–	–
**4**	**–**	**–**	**–**	83	+	+++	+++	+++	+++	78	60	+	–
**5**	**–**	**–**	**–**	80	+	+++	+++	+++	+++	82	78	–	–
**6**	**–**	**–**	**–**	87	+	++	+++	+++	+++	83	63	–	–
**7**	**–**	**–**	**–**	77	+	+++	+++	+++	+++	74	65	+	+
**8**	**–**	**–**	**–**	82	+	+++	+++	+++	++	67	69	–	–
**9**	**–**	**–**	**–**	70	+	+++	+++	+++	+++	63	67	–	–
**10**	**–**	**–**	**–**	86	+	++	+++	+++	+++	73	65	–	+
**11**	**–**	**–**	**–**	79	+	+++	+++	+++	+++	81	71	–	–
**12**	**–**	**–**	**–**	85	+	+++	+++	+++	+++	76	64	+	–
**13**	**–**	**–**	**–**	81	+	+++	+++	+++	+++	83	65	–	–
**14**	**–**	**–**	**–**	84	+	+++	+++	+++	+++	67	60	–	–
**15**	**–**	**–**	**–**	81	+	++	+++	+++	+++	69	64	–	+
**16**	**–**	**–**	**–**	75	+	+++	+++	+++	++	78	63	–	–
**17**	**–**	**–**	**–**	71	–	++	+++	+++	++	71	72	–	–
**18**	**–**	**–**	**–**	78	+	+++	+++	+++	+++	71	71	–	–
**19**	**–**	**–**	**–**	76	+	+++	+++	+++	+++	75	70	–	+
**20**	**–**	**–**	**–**	80	+	+++	+++	+++	+++	70	57	+	–
**21**	**–**	**–**	**–**	76	+	+++	+++	+++	++	82	71	–	–
**22**	**–**	**–**	**–**	77	+	+++	+++	+++	+++	81	67	–	–
**23**	**–**	**–**	**–**	70	–	++	+++	+++	+++	71	80	–	+
**24**	**–**	**–**	**–**	74	+	+++	+++	+++	+++	80	75	+	–
**25**	**–**	**–**	**–**	74	+	+++	+++	+++	+++	74	65	–	–
**26**	**–**	**–**	**–**	80	+	+++	+++	+++	+++	68	70	+	–

The immunostaining of sections was semi-quantitatively scored as: “-” – negative expression (0% immunoreactive cells); “+” – low expression (5–25% immunoreactive cells); “++” – moderate expression (26–50% immunoreactive cells); “+++” – high expression (>50% immunoreactive cells). Only the p53 nuclear expression pattern was considered as positive or negative staining. Ki-67, Hsp70 and Hsp90 expression were scored in percentage

**Figure 3:**
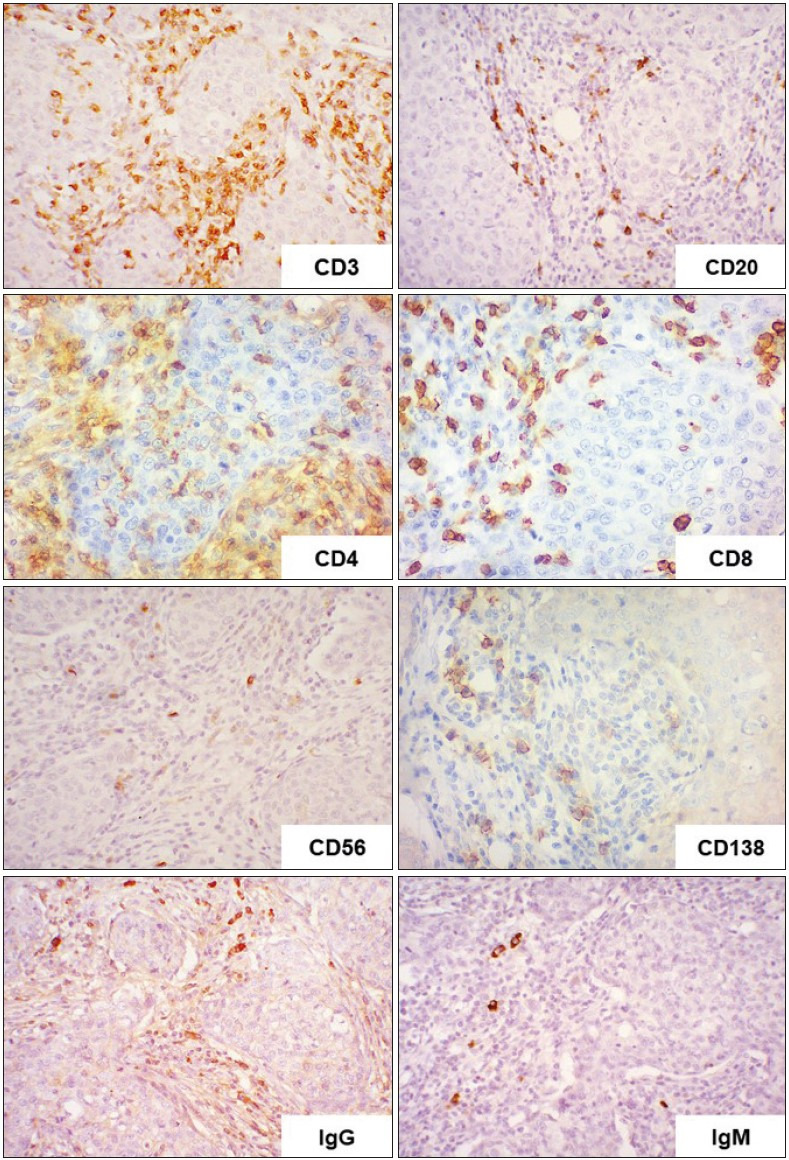
IBC-NST with medullary pattern: Immunohistochemical study of CD20, CD3, CD4, CD8, CD56, CD138, IgG, and IgM ; x400.

**Figure 4:**
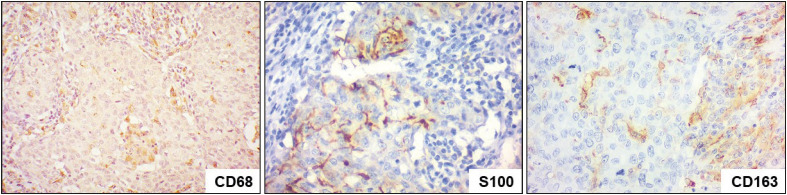
IBC-NST with medullary pattern: Immunohistochemical study of CD68, S100, and CD163; x400.

We focused on studying the qualitative composition of the tumor microenvironment of IBC-NST with medullary pattern. Immunohistochemical analysis of the expression of CD20 (B-lymphocytes), CD3 (T-lymphocytes), CD4 (T-helper cells), CD8 (T-killer cells), CD56 (natural killer cells), CD138 (plasmocytes), CD68, S100, and CD163 (macrophages), MPO (granulocytes), IgG, and IgM revealed the specific features of the immune infiltrate (Figure 2 Figure 3 Figure 4).

The tumor immune-infiltrate microenvironment mainly comprised T-lymphocytes (44% of immune cells) with a relatively low number of B-lymphocytes, macrophages, plasmocytes, and granulocytes (13%, 22%, 9%, and 3% of immune cells, respectively). T-helper cells (CD4+ cells), with a relatively small number of T-killer cells (CD8+ cells) and NK cells (CD56+ cells), were observed among the T-lymphocytes (Figure 3). The tumor microenvironment contained single and grouped plasmocytes, which reached a prevalence of about 8% of the immune-cell infiltrate in some cases. Most B-lymphocytes and plasmocytes showed IgG expression, and some showed IgM expression.

Macrophages (CD68+ cells) were present in significant numbers both among tumor cells and in the immune infiltrate (Figure 4). It should be noted that their active forms (S100+ cells) were mainly localised among the cancer cells. A significant population of M2 macrophages (CD163+ cells) was identified, and they were diffused throughout all the components of the neoplastic tissue.

The scores for each cell type in the tumor microenvironment showed the following ratios: CD3+:CD20+:CD138+:CD56+:CD68+:MPO = 0.44: 0.13: 0.09: 0.09: 0.22: 0.03.

## DISCUSSION

The malignancy of tumors is determined by various factors, including morphological and molecular-genetic features of the affected tissues (2,5,7,8). The qualitative composition of the tumor microenvironment is of particular interest (5,6,9,10). Here we focused on IBC-NST with medullary pattern, which has unique favourable prognostic features with simultaneous high-grade tumor growth (2,5–7).

Our study investigated the immunohistochemical features of IBC-NST with medullary pattern to explain the inconsistency between the morphological and clinical characteristics of this tumor type. We found that the cancer cells had pronounced proliferative activity (Kі-67 expression), and blocked both natural and drug-induced apoptosis (p53 and Bcl-2 expression), as well as the expression of unfavourable proteins (EGFR, Hsp70, Hsp90, and PD-L1) that induce progression of the neoplastic process (8,9,11–13). At the same time, the cells were ER and PR-negative. In contrast, most of the tumor cells had no HER2/neu expression, low VEGF and MMP1 expression (weak expression in less than 25% of tumor cells), and high E-cadherin expression (strong expression in more than 50% of tumor cells), which indicated a favourable course of the carcinoma (14–17).

Our results showed pronounced variability in the qualitative composition of the tumor microenvironment of IBC-NST with medullary pattern. This was represented mainly by TILs, which were predominantly T-lymphocytes and, in particular, CD4+ cells. Simultaneously, significant numbers of CD8+ T-cells, NK cells, В-lymphocytes, and plasmocytes were found in the immune infiltrate. Furthermore, the neoplastic tissue was infiltrated with a significant quantity of macrophages. Notably, most of the activated macrophages (S100+ cells) were in close contact with cancer cells. At the same, a significant number of M2 macrophages (CD163+ cells) were found in the tumor microenvironment, which could have an unfavourable adverse effect on the course of the neoplastic process (5,9,18,19).

**Figure 5:**
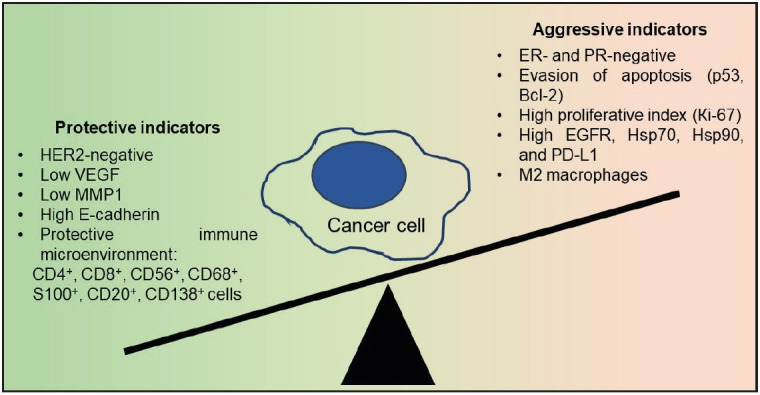
Interaction of protective and aggressive factors in IBCNST with medullary pattern.

From the abovementioned findings, we concluded that the unique course of IBC-NST with medullary pattern is determined by several factors (Figure 5). This tumor type was characterized by a combination of both aggressive and favourable immunophenotypic features of cancer cells and the immune microenvironment. On the one hand, ER and PR were absent in the tumor tissue; there was a high proliferative index; overexpression of p53, Bcl-2, EGFR, hsp70, hsp90, and PD-L1; and significant numbers of M2 macrophages. On the other hand, there were insufficient amounts of pro-metastatic proteins (VEGF and MMP1); an absence of HER2/neu expression; and the severe adhesive capacity of tumor cells prevented local and remote tumor spread. The activation of cellular (macrophages, CD4+ and CD8+ T-cells, and NK cells) and humoral (activated macrophages, B-lymphocytes, CD4+ T-cells, plasmocytes, and IgG synthesis) local immune responses limited the spread of the disease.

In IBC-NST with medullary pattern, the favourable factors (protection indicators) mostly surpassed the adverse influences. This led to a favourable prognosis for the course of the malignant process, despite the numerous negative morphological and immunohistochemical features of the parenchymal and stromal tumor components.

In summary, IBC-NST with medullary pattern has many prognostically unfavourable morphological and immunohistochemical characteristics, which are balanced by the pronounced protective properties of the tumor cells and the qualitative traits of the tumor microenvironment. This leads to a favourable course for this carcinoma despite the relatively adverse features of the cancer cells.

## Conflict of Interest

The authors declare that they have no competing interests.

## Informed Consent

Written informed consent was obtained from patients who participated in this study.
